# Enhanced Sensitivity of MoTe_2_ Chemical Sensor through Light Illumination

**DOI:** 10.3390/mi8050155

**Published:** 2017-05-12

**Authors:** Zhihong Feng, Yuan Xie, Enxiu Wu, Yuanyuan Yu, Shijun Zheng, Rui Zhang, Xuejiao Chen, Chonglin Sun, Hao Zhang, Wei Pang, Jing Liu, Daihua Zhang

**Affiliations:** State Key Laboratory of Precision Measurement Technology and Instruments, School of Precision Instrument and Opto-Electronics Engineering, Tianjin University, No. 92 Weijin Road, Tianjin 300072, China; zhfeng@tju.edu.cn (Z.F.); yuanxie@tju.edu.cn (Y.X.); enxiuwu@tju.edu.cn (E.W.); yuanyuanyu@tju.edu.cn (Y.Y.); zhengshj04@tju.edu.cn (S.Z.); ruizhangmems@tju.edu.cn (R.Z.); chenxuejiao@tju.edu.cn (X.C.); haozhang@tju.edu.cn (H.Z.); weipang@tju.edu.cn (W.P.); jingliu_1112@tju.edu.cn (J.L.)

**Keywords:** MoTe_2_, transition metal dichalcogenides, light illumination, sensitivity, chemical sensor

## Abstract

Two-dimensional (2D) transition metal dichalcogenides (TMDCs) semiconducting materials have recently attracted wide attention and been regarded as promising building blocks for chemical sensors due to their high surface-to-volume ratio. However, their low response hinders the realization of high-performance 2D TMDCs chemical sensors. Here, we demonstrate the improvement of sensing performance of molybdenum ditelluride (MoTe_2_) gas sensor through continuous light illumination. The dependence of sensing performance on the energy of photons and light intensity is systematically studied. The response to NH_3_ is dramatically enhanced by more than 25 times under 254 nm ultraviolet (UV) light illumination with intensity of 2.5 mW/cm^2^. Moreover, a remarkable low detection limit of 3 ppb is achieved, which is improved by 80 times compared with that in dark condition. The results demonstrate that light illumination is a promising method to improve the sensitivity of 2D TMDCs chemical sensors.

## 1. Introduction 

Detecting ultra-low concentration of toxic and harmful gases has become more and more important in many promising applications such as environmental monitoring, industrial manufacturing and diagnostic processes, etc. [1]. In particular, semiconductor chemical sensors have attracted considerable attention owing to their low cost, ease of integration and simple sensing mechanism based on the conductance change upon gas exposure [2]. Over the past few decades, metal oxide semiconductor sensors have delivered satisfactory sensitivity in many cases [3,4,5], however, they require very high temperature (generally above 200 °C) to activate the chemisorption of atmospheric oxygen on the metal oxide surface, which raises several thermal-related issues and restricts their applications in oxygen-free environments or hazardous environments containing flammable gas species. Therefore, highly-sensitive chemical sensors which can operate at room temperature are highly desired. Recently, semiconducting two-dimensional (2D) transition metal dichalcogenides (TMDCs) have been widely studied and considered as promising gas-sensing materials due to their high surface-to-volume ratio and favorable surface energy level for gas adsorption [6,7,8,9,10]. Besides, researchers have also developed various approaches, for instance, surface functionalization [11] and using vertically aligned structure [12] to further enhance the sensitivity of 2D TMDCs based chemical sensors. However, the results show limited improvement, which is usually less than 10 times. Illuminating sensors with light is another effective way to dramatically improve the performance of semiconductor chemical sensors [13,14,15]. However, few have systematically investigated the influence of light illumination on the performance of 2D TMDCs based sensors. 

Molybdenum ditelluride (MoTe_2_), a new addition to the class of 2D TMDCs, has a smaller band gap of ~1.0 eV compared with other TMDCs, which extends the photo detection range of TMDCs based phototransistor from the visible to near-infrared range [16]. Recent experimental investigations show that MoTe_2_ photodetector has a high photoresponsivity and a fast photoresponse in broad spectral range [17,18]. In the view of growing attention on MoTe_2_ for possible optoelectronic device applications, it is also important to investigate the influence of light illumination on the sensing performance of MoTe_2_ based chemical sensors. 

In this work, we report the improvement of gas-sensing properties of MoTe_2_ chemical sensor through light illumination. The sensitivity of MoTe_2_ sensor for NH_3_ detection was enhanced by more than 25 times and the detection limit as low as 3 ppb was achieved under continuous ultraviolet (UV) light illumination. The influences of wavelength and intensity of light on the improvement of the sensing properties have also been systematically studied.

## 2. Materials and Methods

**Figure 1:**
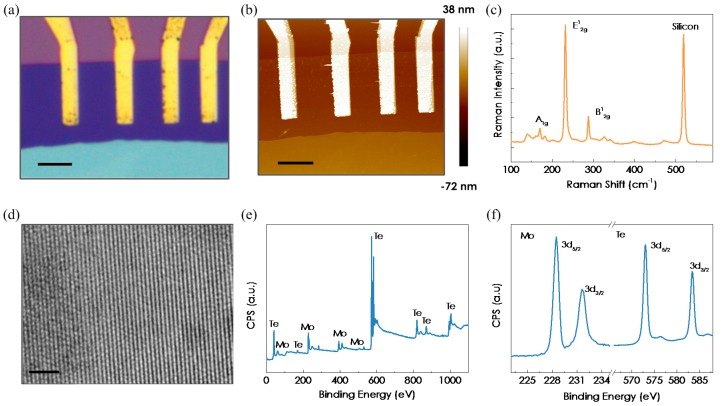
(**a**) Optical microscope image of the MoTe_2_ field-effect transistor (FET) on top of SiO_2_/Si substrate with Ti/Au electrodes. Scale bar is 5 μm. (**b**) Atomic force microscopy (AFM) topography image of the MoTe_2_ FET. Thickness of the MoTe_2_ is 3.4 nm. Scale bar is 5 μm. (**c**) Raman spectra of the few-layered MoTe_2_. The characteristic Raman-active modes of an A_1g_ peak at 170 cm^−1^, a E^1^_2g_ peak at 231 cm^−1^ and a B^1^_2g_ peak at 287 cm^−1^ are clearly observed. (**d**) High-resolution transmission electron microscopy (TEM) image of a typical exfoliated MoTe_2_ film. Scale bar is 2 nm. (**e**) X-ray photoelectron spectroscopy (XPS) spectrum of as-used MoTe_2_ crystal. (**f**) High resolution Mo 3d and Te 3d XPS spectra of the MoTe_2_ crystal. 3d_5/2_ Mo, 3d_3/2_ Mo, 3d_5/2_ Te and 3d_3/2_ Te peaks are clearly observed.

Few-layered MoTe_2_ flakes were prepared by mechanical exfoliation of a MoTe_2_ bulk crystal on n+ doped silicon substrate covered with 285 nm of thermally grown SiO_2_. The tape residues after exfoliation were removed by dipping the substrate along with flakes in acetone for 10 min. The electrical contacts on MoTe_2_ were fabricated by lift-off process using electron beam lithography and subsequent electron beam evaporation of 20/60 nm Ti/Au. Figure 1a shows the optical microscope image of a typical fabricated MoTe_2_ field-effect transistor (FET) device. Thickness of the MoTe_2_ flake is 3.4 nm, as confirmed by the atomic force microscopy (AFM, Bruker, Santa Barbara, CA, USA) measurement shown in Figure 1b. MoTe_2_ has two stable crystal phases: semiconducting 2H (hexagonal) and metallic 1T′ (distorted octahedral) phases [19]. Density functional theory (DFT) calculations revealed that molecular adsorption favors semiconducting 2H phase other than 1T′ metallic phase [20]. So it is important to determine the exact crystal structure of the MoTe_2_ flakes we used. Figure 1c shows the Raman spectra (532 nm excitation wavelength) for the exfoliated few-layered MoTe_2_. The characteristic Raman-active modes of the out-of-plane A_1g_ mode at 170 cm^−1^, prominent in-plane E^1^_2g_ mode at 231 cm^−1^ and the out-of-plane B^1^_2g_ mode at 287 cm^−1^ are clearly observed. These Raman features are signature peak positions of 2H phase MoTe_2_ and in good agreement with the results reported previously [19]. The crystallographic structure of the MoTe_2_ was further characterized by high-resolution transmission electron microscopy (TEM, JEOL, Tokyo, Japan). Figure 1d shows a typical TEM image of as-exfoliated MoTe_2_ film. We could clearly observe a high level of crystallinity and hexagonal symmetry. X-ray photoelectron spectroscopy (XPS, PerkinElmer, Waltham, MA, USA) was utilized to determine the elemental composition and bonding types of the MoTe_2_ flakes. The XPS spectrum displayed in Figure 1e reveals the presence of Mo and Te elements in the flakes. Peaks are observed at 228.4, 231.5, 573 and 583.4 eV, corresponding to the Mo 3d_5/2_, Mo 3d_3/2_, Te 3d_5/2_ and Te 3d_3/2_, respectively, as shown in Figure 1f. These features are consistent with previous results from 2H MoTe_2_ [16].

## 3. Results and Discussion

**Figure 2:**
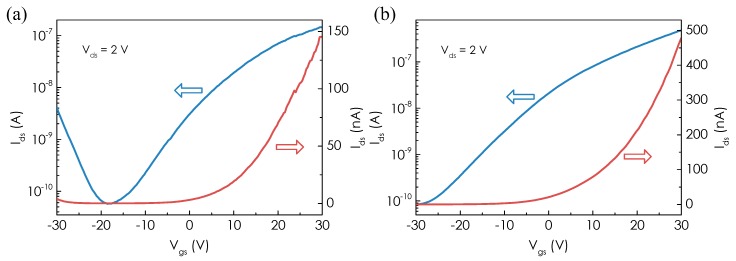
(**a**) Transfer characteristics of the MoTe_2_ FET before UV illumination, *V*_ds_ = 2 V. (**b**) Transfer characteristics of the MoTe_2_ FET after 2 h UV illumination, *V*_ds_ = 2 V.

We then investigate the effect of UV light illumination on the electrical performance of the MoTe_2_ FET device in pure N_2_ (99.9999%) environment. The wavelength of UV light source is 254 nm and the intensity is 2.5 mW/cm^2^. Figure 2a shows the transfer characteristics of the MoTe_2_ FET device in both linear and semi-log scales under dark condition. The device shows ambipolar behavior with a minimum current around *V*_gs_ = −18 V. The *I*_ds_ increases dramatically by 2 × 10^3^ when *V*_gs_ increases from −18 V to 30 V, indicating electron accumulation in the MoTe_2_ channel. When *V*_gs_ decreases from −18 V to −30 V, the *I*_ds_ is dominated by holes, as evidenced by increasing *I*_ds_. The observation is also consistent with previous studies [21]. After UV light illumination for 2 h, we note that the p-type behavior vanishes and the MoTe_2_ FET device behaves as n-type transistor as evidenced in the *I*_ds_–*V*_gs_ curves in Figure 2b. When *V*_gs_ increases from −30 to 30 V, *I*_ds_ increases monotonically by more than 3 orders of magnitude due to accumulation of electrons in the conduction channel. The vanished p-type behavior of the MoTe_2_ FET under UV light illumination can be attributed to the removal of O_2_ molecules from MoTe_2_ flake. This can also be confirmed by the reversible electrical characteristics of one MoTe_2_ FET (Figure S1). The oxygen molecules adsorbed on the surface of MoTe_2_ flake may act as acceptors which induce p-type doping [22].

We then carried out systematic sensing tests of the MoTe_2_ FET toward NH_3_ in both dark and light illumination conditions to characterize its sensing performance at room temperature. Prior to the sensing experiments, the gas chamber and device were purged with N_2_ for 2 h under UV light illumination. N_2_ was used as the carrying gas to dilute the target gases to desired concentrations through flow rate adjustments. The gate electrode is grounded throughout all measurements.

**Figure 3:**
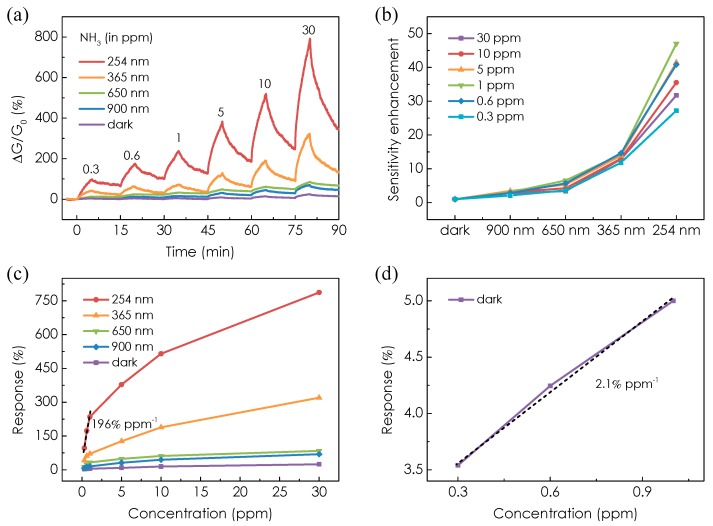
(**a**) Real-time conductance change of the MoTe_2_ sensor upon exposure to different concentrations of NH_3_ in the dark and under light illumination. (**b**) Sensitivity enhancement of the MoTe_2_ sensor as a function of wavelength (photon energy) at different concentrations of NH_3_. (**c**) Response of the MoTe_2_ sensor as a function of NH_3_ concentration in the dark and under light illumination with different wavelength. The calculated slope of the linear fitting (indicated by dashed line) is 196% ppm^−1^ under 254 nm UV light illumination. (**d**) Enlarged part response of the MoTe_2_ sensor as a function of NH_3_ concentration in the dark in (**c**). The calculated slope is 2.1% ppm^−1^.

Figure 3a presents the real time response of the MoTe_2_ sensor toward exposure of NH_3_ from 300 ppb to 30 ppm under both dark and light illumination conditions. The light intensity was kept at 2.5 mW/cm^2^. The device conductance increases monotonically with increasing NH_3_ concentrations for all conditions as NH_3_ molecules adsorption increase the electron concentration in the n-type semiconducting channel. The response is defined as (*G* − *G*_0_)/*G*_0_ × 100%, where *G*_0_ and *G* are the initial conductance and 5 min after NH_3_ exposure, respectively. We note that the response of the MoTe_2_ sensor is enhanced remarkably under light illumination from near-infrared to UV region. For example, the device shows responses ranging from ~4 to 25% under dark condition, whereas it presents larger responses ranging from ~100 to 790% under 254 nm UV light illumination at NH_3_ concentrations of 300 ppb–30 ppm. Therefore, the response is enhanced by more than 25 times by UV light illumination. The response values obtained under UV light illumination are well above the results from 2D TMDCs chemical sensors for NH_3_ detection. For comparison, the study in [9] and [23] demonstrated sensitivity of MoSe_2_ and WS_2_ sensors from a few to tens of ppm concentrations. It is noteworthy that for the MoS_2_ sensor with a lower detection limit down to 300 ppb, the response is ~0.04% [24], which is 3 orders lower than the MoTe_2_ sensor. By comparing the data points at different concentrations of NH_3_ under light illumination with different wavelength, we also note that the sensitivity enhancement increases monotonically with reducing wavelength (increasing photon energy), as shown in Figure 3b. The sensitivity improvement is 2–6-fold in the near-infrared to red region, while it increases to more than 10 times in the UV region. The largest sensitivity enhancement is achieved under 254 nm UV light illumination. The enhanced sensitivity under light illumination can be attributed to the following reasons: The presence of defects in MoTe_2_ during the crystal growth process can act as active sites. Density functional theory calculations revealed that the presence of defects strongly increases the binding energy of the molecular interaction on MoTe_2_ surface [25,26]. For example, interaction at the defect sites, such as Te vacancies, yields much larger binding energy between MoTe_2_ and O_2_ (~166 meV) compared to the case of pure MoTe_2_ (~36–64 meV) [25]. The O_2_ molecules initially adsorbed on the surface of MoTe_2_ may occupy the defect sites. Therefore, removal of O_2_ molecules could significantly enhance the sensitivity. However, the thermal energy at room temperature could not desorb O_2_ molecules [27,28]. In this respect, UV illumination can provide the energy for O_2_ desorption through several mechanisms [29,30]. The study in Reference 29 suggested that photo induced desorption strongly depends on the wavelength of light. UV light illumination has the highest efficiency for “cleaning” the nanomaterial surface owing to electron plasmon excitation. Therefore, the best sensitivity was achieved under UV light illumination. By comparing the response time and recovery rate in dark and light illumination conditions (Figure S2), we also note that the response time is not changed too much while the recovery rate is slower under light illumination than that in dark. This can be attributed to the following reason: NH_3_ molecules are mainly adsorbed on the pristine surface of MoTe_2_ due to occupancy of active sites by O_2_ molecules in dark condition. Under light illumination, the active sites are released and the binding energy between NH_3_ and active sites are much higher than the pristine case. On the other hand, we also note that the recovery rate is faster in the UV region than in the near-infrared to red region. Under UV light illumination, the desorption rate of NH_3_ from active sites is increased due to electron plasmon excitation [29] so that a dynamic equilibrium is established between adsorption and desorption processes.

Figure 3c plots the response as a function of NH_3_ concentration in the dark and under light illumination. The response is linear at low concentrations and shows a quasi-saturation behavior as the concentration increases. The linear portion of the curve can be then used to estimate the detection limit, which is typically defined as the concentration level corresponding to a signal-to-noise ratio (SNR) of 3 [31]. Under 254 nm UV light illumination, the detection limit is estimated to be ~3 ppb by comparing the noise level (0.17%) of the sensor to the slope (196% ppm^−1^) of the response at low concentration of NH_3_. This number is 80 times superior to that in the dark, which is estimated to be ~243 ppb according to the slope of the response (Figure 3d). We also note the detection limit of our MoTe_2_ sensor under 254 nm UV light illumination is the record number using 2D TMDCs so far [6,7,9,23,24,32,33] and competitive with other nano-sensors [34,35,36].

**Figure 4:**
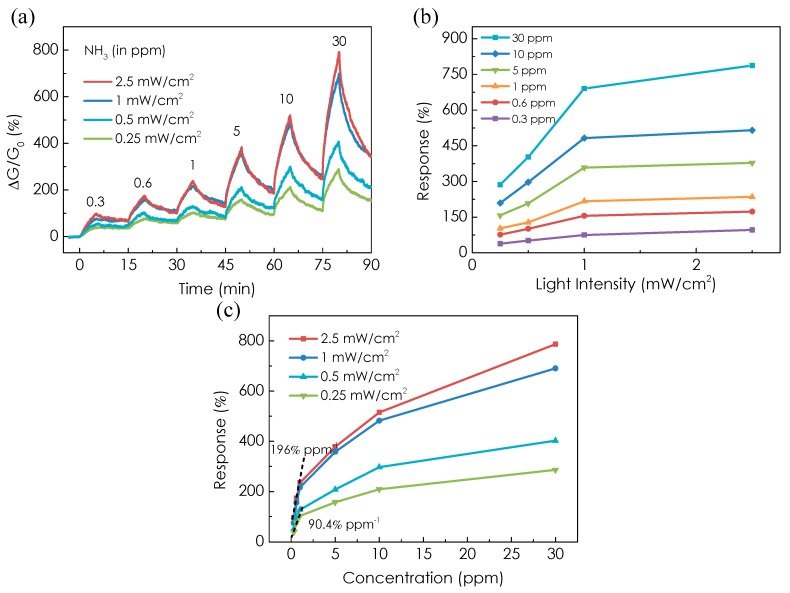
(**a**) Real-time conductance change of the MoTe_2_ sensor upon exposure to different concentrations of NH_3_ under 254 nm UV light illumination with different intensity. (**b**) Response of the MoTe_2_ sensor as a function of light intensity at different concentrations of NH_3_. (**c**) Response of the MoTe_2_ sensor as a function of NH_3_ concentration under 254 nm UV light illumination with different intensity. The calculated slope of the linear fitting (indicated by dashed line) is 90.42% ppm^−1^ and 196% ppm^−1^ at intensity of 0.25 mW/cm^2^ and 2.5 mW/cm^2^, respectively.

Figure 4a shows the dynamic response of the MoTe_2_ sensor to different NH_3_ concentrations (300 ppb–30 ppm) under UV light illumination (254 nm) with intensity ranging from 0.25 to 2.5 mW/cm^2^. We note that the sensing properties of the MoTe_2_ sensor behave similar but the responses are strongly influenced by the light intensity. For example, the response to 30 ppm NH_3_ is ~290% at intensity of 0.25 mW/cm^2^, while it increases up to ~790% at intensity of 2.5 mW/cm^2^. Figure 4b presents the dependence of the response on the UV light intensity. The response increases rapidly when the intensity increases from 0.25 to 1 mW/cm^2^ and then shows slow saturation trend as the intensity increases up to 2.5 mW/cm^2^, the highest intensity we could experimentally achieve with the setup. Generally, the light intensity should have an optimal value for maximum sensitivity as the desorption rate will increase more than the adsorption rate if the intensity is too high [37]; however, we did not observe this phenomena due to the limited power density of our light source. The detection limit is also improved with higher light intensity. The slopes of the response plotted in Figure 4c are 196% ppm^−1^ and 90.4% ppm^−1^ at intensity of 2.5 and 0.25 mW/cm^2^, respectively, from which we can obtain a detection limit of ~3 and 6 ppb, respectively. Therefore, the detection limit is improved by 2-fold as the intensity increases by 10 times.

## 4. Conclusions

In summary, we report a systematic study on the sensitivity enhancement of MoTe_2_ gas sensor through light illumination. Compared with dark conditions, the sensitivity is dramatically improved by light illumination and monotonically dependent on the photon energy. With continuous 254 nm UV light illumination, the response to NH_3_ gas is enhanced by more than 25 times and a detection limit as low as 3 ppb is achieved. It is also found that the enhanced sensitivity is dependent on the light intensity. The response increases from 290 to 790% at 30 ppm NH_3_ when increasing the UV light intensity from 0.25 to 2.5 mW/cm^2^. Our results prove a strong influence of light illumination on the sensitivity of MoTe_2_ sensor and demonstrate the potential of 2D MoTe_2_ as a promising candidate for ultrasensitive chemical sensing applications.

## Supplementary Materials

The following are available online at www.mdpi.com/2072-666X/8/5/155/s1, Figure S1: Transfer characteristics of MoTe_2_ FET (a) before and (b) after UV illumination and (c) after recovery in air. (d–f) Corresponding output characteristics in (a–c). Figure S2: (a) Response time of the MoTe_2_ sensor under different condition. (b) Recovery of the MoTe_2_ sensor under different condition. The response time was defined as the time required to change the conductance after exposure of NH_3_ in a specific range of 90%. The recovery was defined as (*G* − *G*_10min_)/(*G* − *G*_0_) × 100%, where *G*_0_ and *G* are the initial conductance and 5 min after NH_3_ exposure, respectively, 𝐺_10min_ is the device conductance after shutting off the target gas for 10 min.
