# Supplementary material for: Enhanced Sensitivity of MoTe2 Chemical Sensor through Light Illumination
Source: Micromachines (Basel). 2017 May 12;8(5):155. doi: 10.3390/mi8050155 (PMC6189933; doi:10.3390/mi8050155)
Supplement: Supplementary file 1 [file micromachines-08-00155-s001.pdf]

Supplementary Materials: Enhanced Sensitivity of $MoTe_2$ Chemical Sensor through Light Illumination

Zhihong Feng, Yuan Xie, Enxiu Wu, Yuanyuan Yu, Shijun Zheng, Rui Zhang, Xuejiao Chen, Chonglin Sun, Hao Zhang, Wei Pang, Jing Liu, and Daihua Zhang

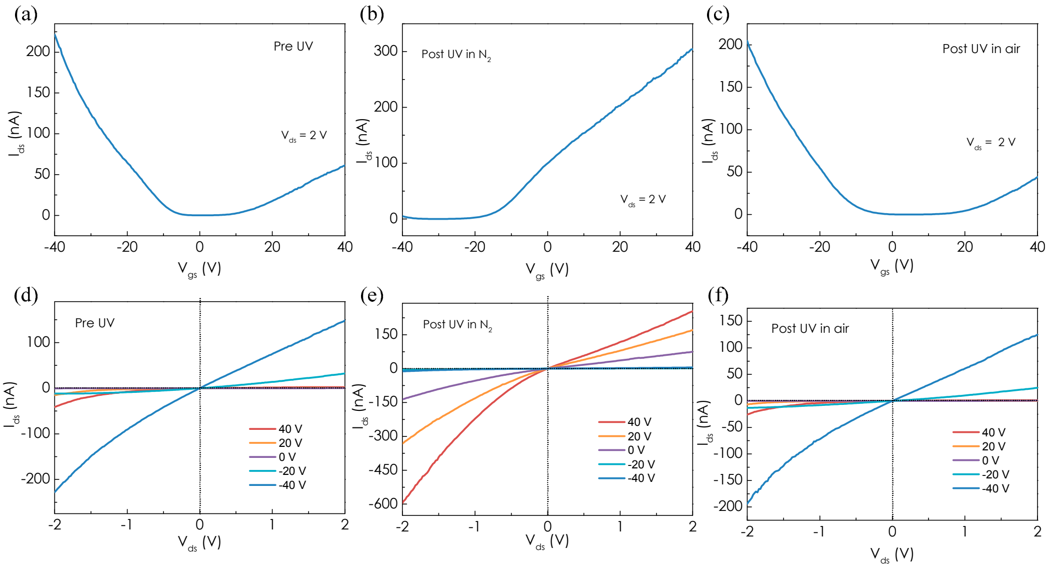

Figure S1. Transfer characteristics of $MoTe_2$ FET (**a**) before and (**b**) after UV illumination and (**c**) after recovery in air. (**d**–**f**) Corresponding output characteristics in (**a**–**c**).

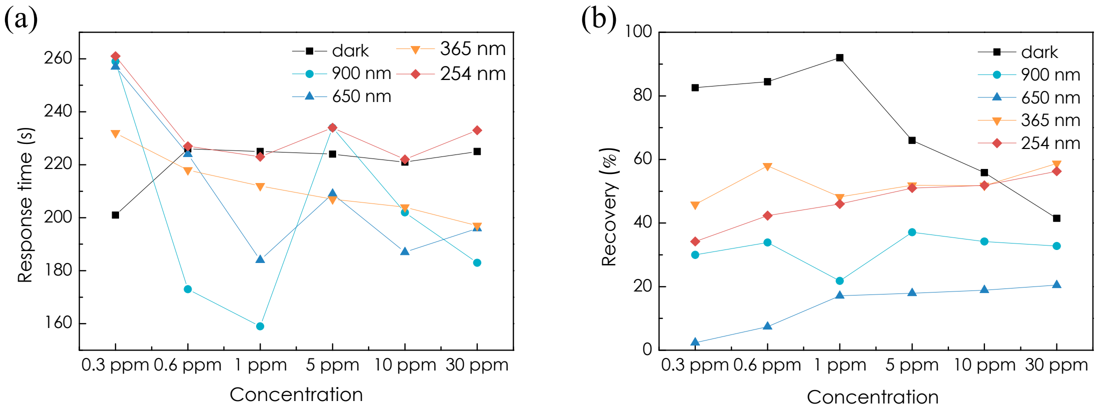

Figure S2. (**a**) Response time of the $MoTe_2$ sensor under different condition. (**b**) Recovery of the $MoTe_2$ sensor under different condition. The response time was defined as the time required to change the conductance after exposure of NH_3 in a specific range of 90%. The recovery was defined as $(G - G_{10min})/(G - G_0) \times 100\%$, where G_0 and G are the initial conductance and 5 min after NH_3 exposure, respectively, $G10min$ is the device conductance after shutting off the target gas for 10 min.
